# ﻿Notes on Dysderidae (Arachnida, Araneae) of Armenia and Iran, with new species and records

**DOI:** 10.3897/zookeys.1172.107112

**Published:** 2023-07-25

**Authors:** Armine Kosyan, Alireza Zamani, Yuri M. Marusik

**Affiliations:** 1 Faculty of Biology, Yerevan State University, Yerevan, Armenia Yerevan State University Yerevan Armenia; 2 Zoological Museum, Biodiversity Unit, FI-20014 University of Turku, Finland University of Turku Turku Finland; 3 Department of Zoology & Entomology, University of the Free State, Bloemfontein 9300, South Africa University of the Free State Bloemfontein South Africa; 4 Altai State University, Lenina Pr., 61, Barnaul, RF-656049, Russia Altai State University Barnaul Russia

**Keywords:** Aranei, Caucasus, red devil spiders, woodlouse spiders

## Abstract

New data are provided on dysderid spiders of Armenia and Iran. *Dysderahaykana***sp. nov.** is described based on male specimens collected in Kotayk and Lor provinces, central and northern Armenia. The female of *Dysderanakhchivanica* Beydizade, Shafaie & Guseinov, 2018 is described for the first time, and the species is newly recorded from Armenia. Furthermore, new distribution data are provided for *Harpacteaarmenica* Dunin, 1989, including the first record of the species from Iran. Photographs and a distribution map are provided for all three species.

## ﻿Introduction

The spider family Dysderidae C.L. Koch, 1837 currently comprises 612 extant species in 25 genera distributed in the Western Palaearctic (WSC 2023). The dysderid fauna of the Caucasus is relatively well studied thanks to the publication of several large-scale, family-level revisions and smaller taxonomic contributions (e.g., Charitonov 1956; Dunin 1982, 1987, 1990, 1991, 1992). However, the Armenian dysderids, with only 11 currently known species (Otto 2022), remain poorly documented, especially when compared to adjacent countries of Azerbaijan (30 species; Otto 2022), Georgia (33 species; Otto 2022), Iran (15 species; Zamani et al. 2023), and Turkey (69 species; Danışman et al. 2023). This paper aims to contribute to the knowledge of dysderid diversity in this region by providing new taxonomic and faunistic data on three species occurring in Armenia and Iran.

## ﻿Materials and methods

Photographs of specimens and their copulatory organs were obtained using an Olympus Camedia E‐520 camera attached to an Olympus SZX16 stereomicroscope, and a JEOL JSM-5200 scanning electron microscope. Digital images of different focal planes were stacked with Helicon Focus™ v. 8.1.1. Illustrations of internal genitalia were made after digesting tissues off in a 10% KOH aqueous solution. Body measurements exclude the chelicerae and spinnerets. Leg segments were measured on the dorsal side. Measurements and characters of the palp used in the diagnoses are based on the retrolateral view. Measurements of legs are listed as: total length (femur, patella, tibia, metatarsus, tarsus). All measurements are given in millimetres. The map was prepared using SimpleMappr (Shorthouse 2010).

**Abbreviations: Eyes: AME** – anterior median eye, **PLE** – posterior lateral eye, **PME** – posterior median eye. **Spination: d** – dorsal, **Fe** – femur, **Mt** – metatarsus, **Pa** – patella, **pl** – prolateral, **rl** – retrolateral, **Ti** – tibia, **v** – ventral.

**Depositories: MHNG** – Muséum d’histoire naturelle, Genève, Switzerland (P.J. Schwendinger, L. Monod); **ZMMU** – Zoological Museum of the Moscow State University, Russia (K.G. Mikhailov).

## ﻿Taxonomy

### ﻿Family Dysderidae C.L. Koch, 1837

**Subfamily Dysderinae C.L. Koch, 1837**

**Genus *Dysdera* Latreille, 1804**

#### ﻿ Dysdera haykana sp. nov.

Taxon classificationAnimaliaAraneaeDysderidae

901AE16D-09FE-5966-B6A2-22B92D083C00

https://zoobank.org/F47E0340-1B68-488A-8B19-4291FADF47F7

**Figure 1.**
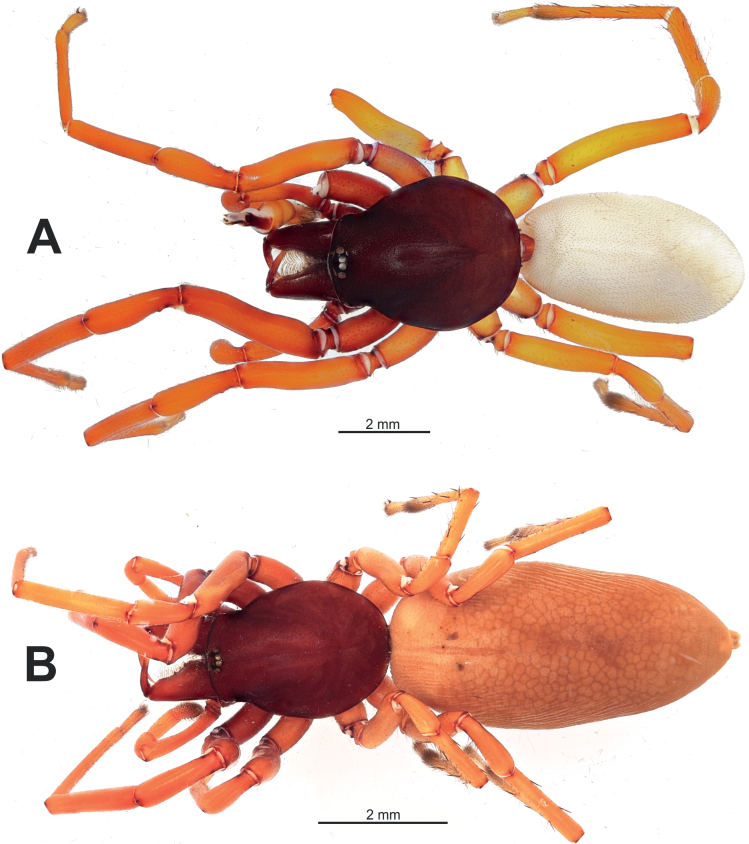
Habitus of the holotype male of *Dysderahaykana* sp. nov. (**A**) and the female of *D.nakhchivanica* (**B**), dorsal.

**Figure 2.**
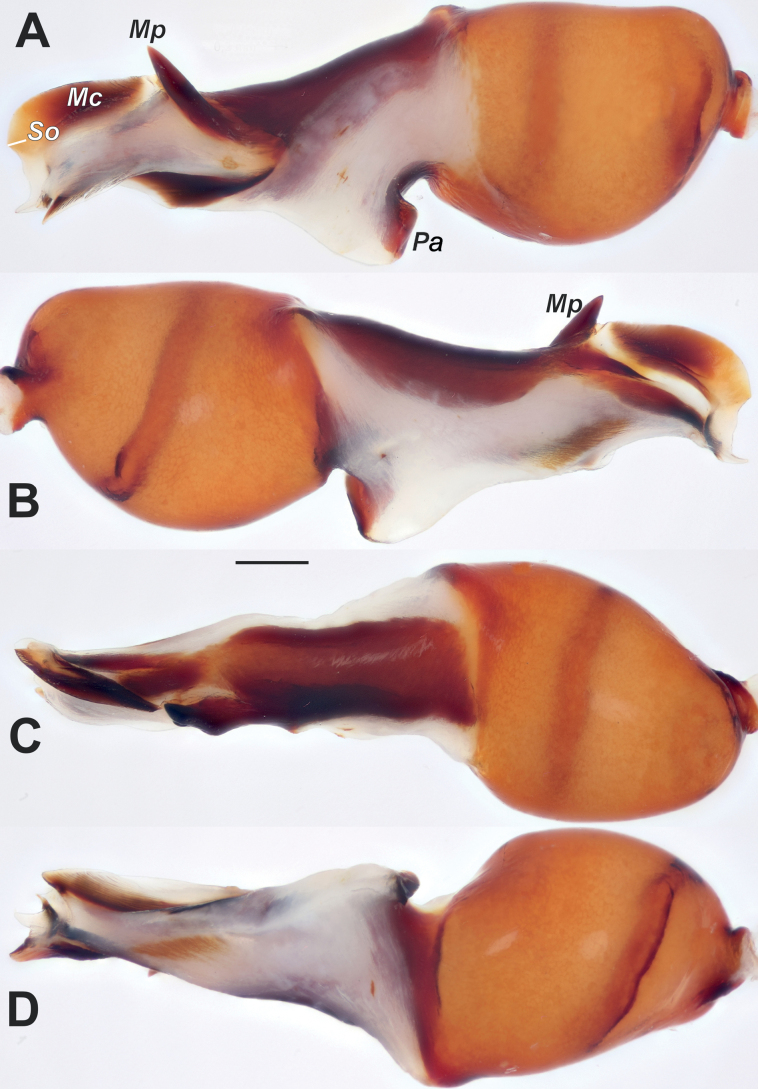
Male of *Dysderahaykana* sp. nov., bulb. **A** retrolateral **B** prolateral **C** dorsal **D** ventral. Abbreviation: *Mc* – median crest, *Mp* – median process, *Pa* – posterior apophysis, *So* – spine-like outgrowth. Scale bar: 0.2 mm.

**Figure 3.**
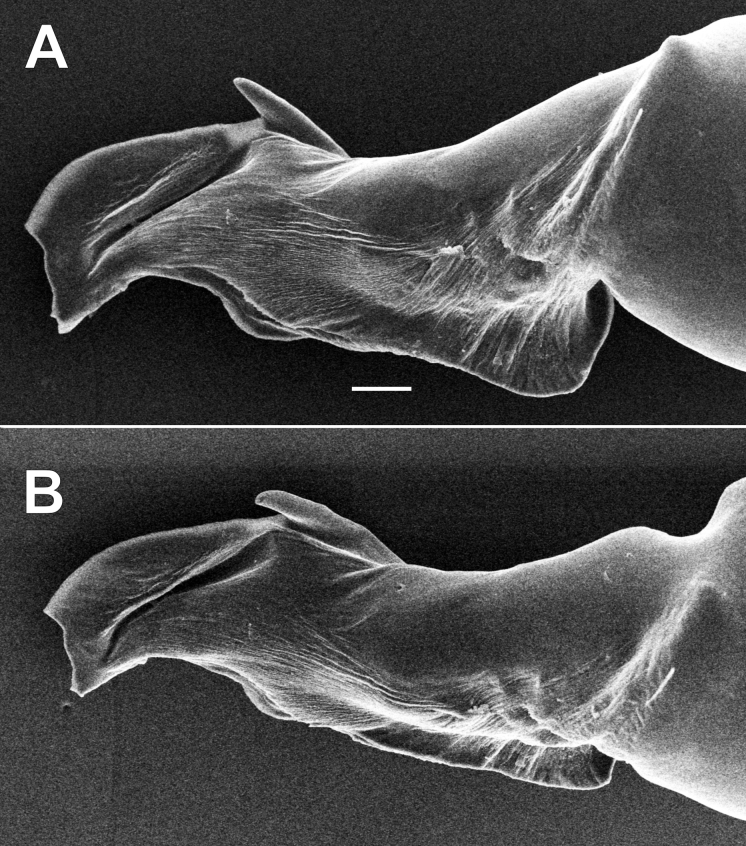
Male of *Dysderahaykana* sp. nov., bulb. **A** prolateral **B** dorso-prolateral. Scale bar: 0.1 mm.

Figs 1A , 2A–D , 3A, B

##### Type materials.

***Holotype*** ♂ (ZMMU), Armenia: *Lori Province*: Mets Parni, 40°49'15.4"N, 44°04'33.2"E, 30.04.2022 (A. Kosyan). ***Paratypes***: 1♂ (ZMMU), same data as for the holotype; 1♂ (ZMMU), *Kotayk Province*: env. of Solak Vill., 40°28'34"N, 44°42'57"E, 14.05.2021 (Y.M. Marusik).

##### Etymology.

The specific epithet is an adjective referring to Hayk Nahapet (in Armenian: Հայկ), the legendary patriarch and founder of the Armenian nation.

##### Diagnosis.

The new species belongs to the *asiatica* species-group and is most similar to *D.asiatica* Nosek, 1905 (see Deeleman-Reinhold and Deeleman 1988: figs 226, 227) and *D.ukrainensis* Charitonov, 1956 (as illustrated by Charitonov 1956: fig. 16) in possessing a spine-like median process (*Mp*) (vs either lacking or of a different shape (with 2 or more teeth) in other species). *Dysderahaykana* sp. nov. can be distinguished from both species by the almost indistinct spine-like outgrowth (*So*) on the median crest (vs distinct). From *D.ukrainensis*, it also differs by having median crest (*Mc*) ca 2.5 times longer than high (vs as long as high), the median process extending the median crest (vs not extending), and the relatively larger posterior apophysis (*Pa*) (cf. Fig. 2A and Charitonov 1956: fig. 16).

##### Description.

**Male** (Holotype). Habitus as in Fig. 1A. Total length 9.22. Carapace 4.16 long, 3.33 wide. Eye sizes: AME 0.22, PME 0.16, PLE 0.19. Carapace and chelicerae dark reddish; carapace slightly lighter posteriorly. Sternum, labium, and maxillae reddish. Legs yellowish orange. Abdomen light beige, without any pattern. Spinnerets uniformly beige. Measurements of legs: I: 11.00 (3.09, 2.11, 2.59, 2.49, 0.72), II: 10.91 (3.13, 1.90, 2.52, 2.69, 0.67), III: 8.23 (2.48, 1.34, 1.52, 2.26, 0.63), IV: 10.85 (3.16, 1.80, 2.23, 2.91, 0.75). Spination: I, II: no spines; III: Ti: 2pl, 1rl, 2v; Mt: 3pl, 3rl, 6v. IV: Fe: 1d; Ti: 2pl, 2rl, 2v; Mt: 2d, 3pl, 3rl, 4v.

Palp as in Figs 2A–D, 3A, B; bulb 3 times longer than its maximal width; psembolus 1.5 times longer than tegulum; tegulum 1.27 times longer than wide; median crest (*Mc*) elongate, ca 2.5 times longer than high, with almost indistinct spine-like outgrowth (*So*), middle part of psembolus with spine-like process (*Mp*), its tip extending dorsal margin of median crest.

**Female.** Unknown.

##### Note.

The record of *D.ukrainensis* in central Georgia (cf. Otto 2022) may belong to this species. Kovblyuk et al. (2008) redescribed the types of *D.ukrainensis*, although their figures of the lectotype male differs from those provided in Charitonov (1956) in the shape of the median process. Moreover, according to Kovblyuk et al. (2008), the median process can vary in shape and may be bifurcated on the tip.

##### Distribution.

Known only from the listed localities in Kotayk and Lori provinces, central and northern Armenia (Fig. 10).

#### ﻿ Dysdera nakhchivanica

Taxon classificationAnimaliaAraneaeDysderidae

Beydizade, Shafaie & Guseinov, 2018

040D3138-FBA0-5AAD-96F7-75B21C5479C7

**Figure 4.**
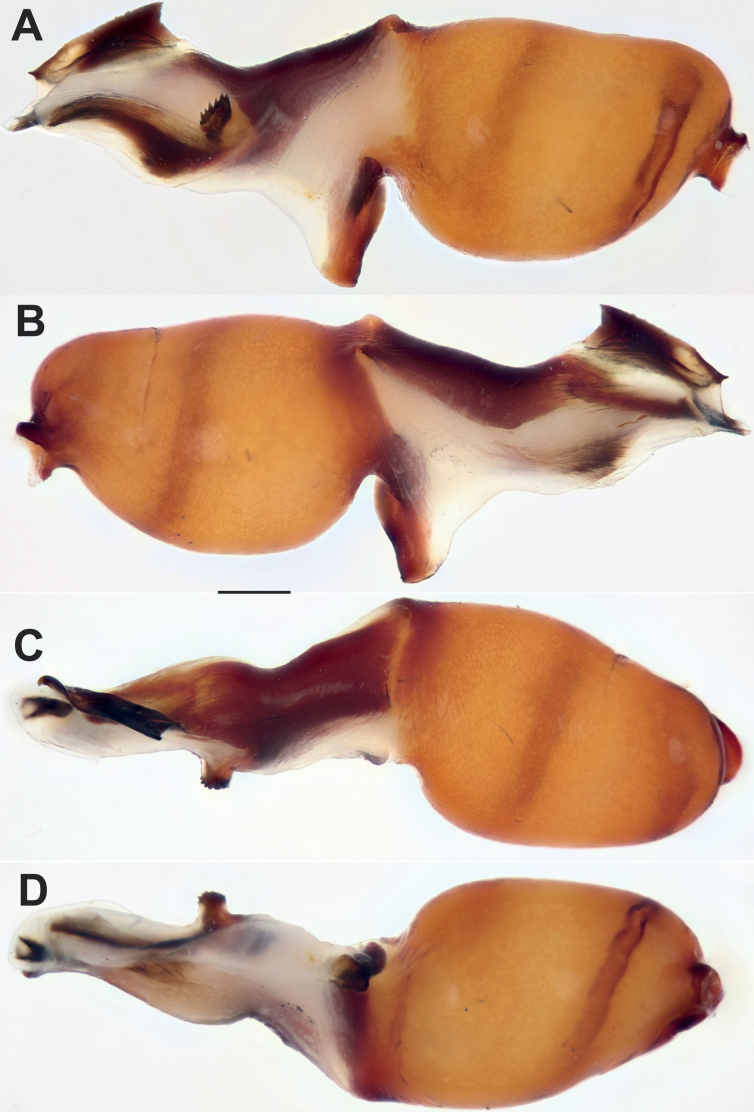
Male of *Dysderanakhchivanica*, bulb. **A** retrolateral **B** prolateral **C** dorsal **D** ventral. Scale bar: 0.2 mm.

**Figure 5.**
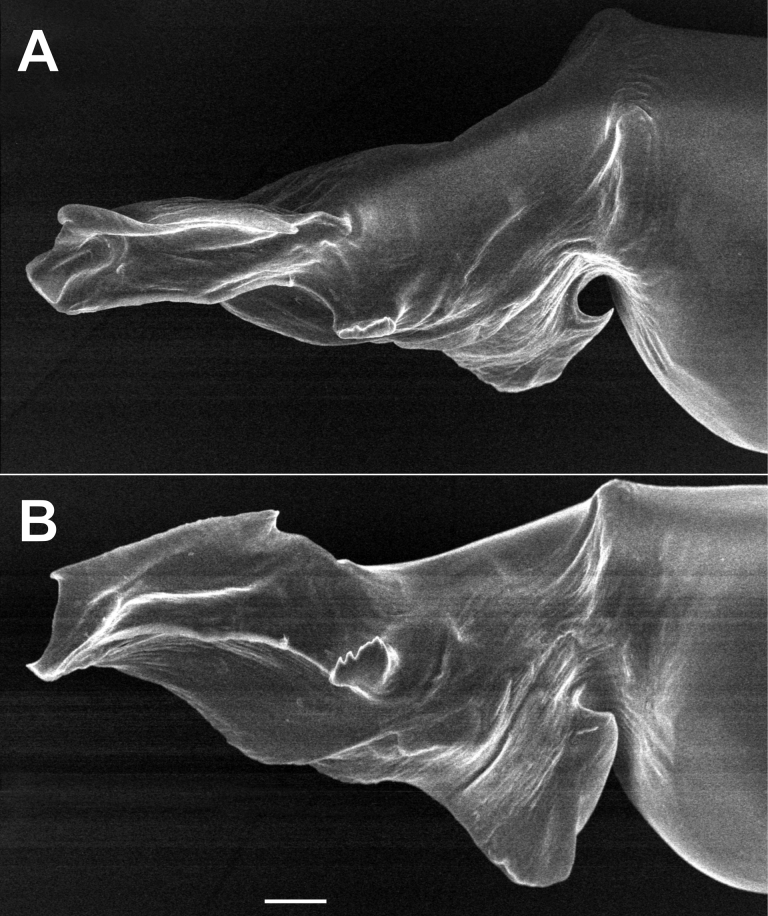
Male of *Dysderanakhchivanica*, bulb. **A** dorsal **B** retrolateral. Scale bar: 0.1 mm.

**Figure 6.**
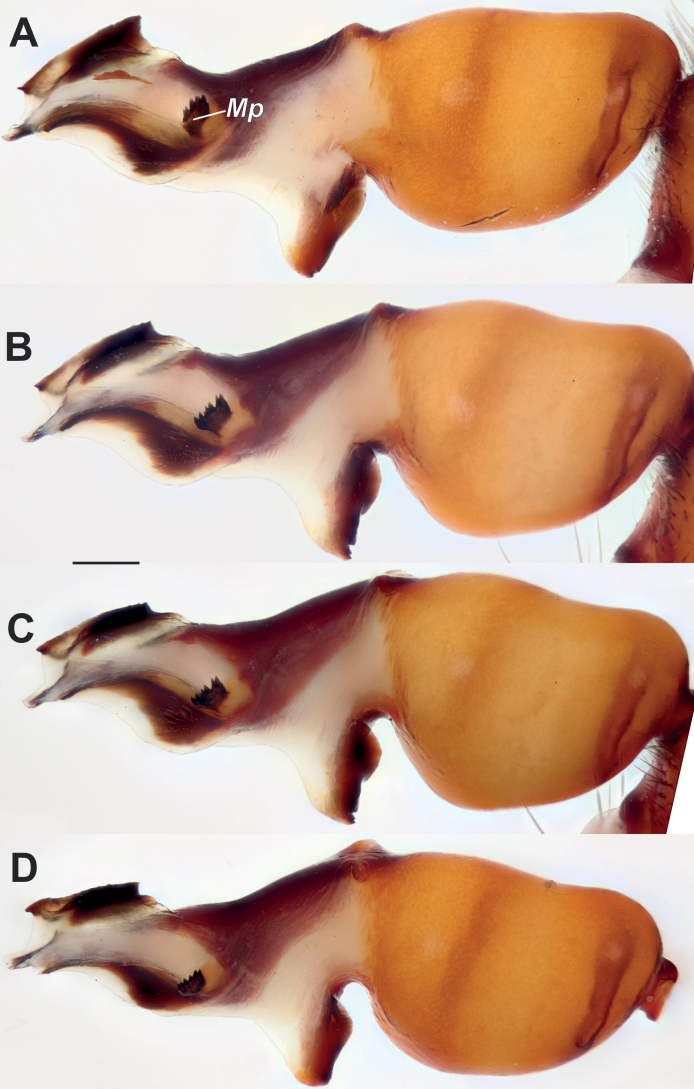
Males of *Dysderanakhchivanica*, bulbs of four specimens in retrolateral view, showing variations. Abbreviation: *Mp* – median process. Scale bar: 0.2 mm.

**Figure 7.**
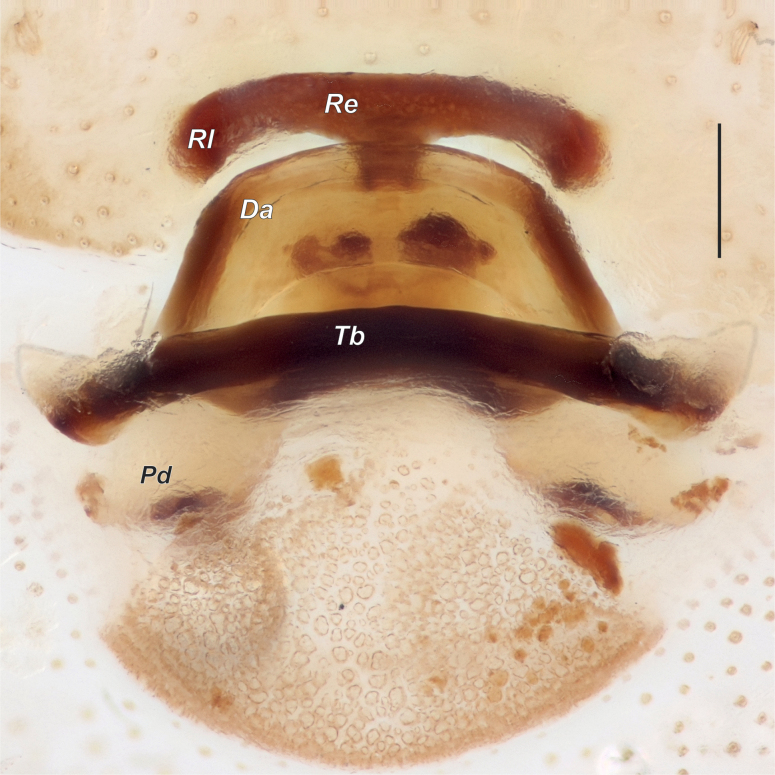
Female of *Dysderanakhchivanica*, endogyne, dorsal. Abbreviations: *Da* – dorsal arch, *Pd* – posterior diverticulum, *Re* – receptacle, *Rl* – receptacular lateral edge, *Tb* – transverse bar. Scale bar: 0.2 mm.

Figs 1B , 4A–D , 5A, B , 6A–D , 7

Dysdera nakhchivanica Beydizade et al., 2018: 1112, figs 1–4, 7–9 (♂).

##### Material examined.

Armenia: *Vayots Dzor Province*: 1♂ 1♀ 2 juv. (ZMMU), env. of Gnishik Vill., 39°40'18"N, 45°17'40"E, ca 2030 m, 11.05.2021 (Y.M. Marusik); 3♂ 3♀ 6 juv. (ZMMU), Shatin, 39°50'N, 45°19'E, 9.05.2021 (Y.M. Marusik).

##### Diagnosis.

For the male, see Beydizade et al. (2018). The female of *D.nakhchivanica* is most similar to that of *D.collucata* Dunin, 1991, a species restricted to southernmost Armenia. The two species have tips of receptacular lateral edges (*Rl*) bent posteriorly (vs not bent or directed anteriorly in other species) and have membranous posterior diverticula (*Pd*) unknown in other *Dysdera* species in the Caucasus or adjacent regions. The female of *D.nakhchivanica* differs from that of *D.collucata* by the relatively longer (i.e., three times wider than long) and distinctly trapezoidal dorsal arch (*Da*).

##### Description.

**Male.** See Beydizade et al. (2018).

**Female.** Habitus as in Fig. 1B. Total length 11.87. Carapace 4.12 long, 3.10 wide. Eye sizes: AME 0.16, PME 0.15, PLE 0.17. Carapace and chelicerae dark reddish; carapace slightly lighter posteriorly. Sternum, labium, and maxillae reddish. Legs yellowish orange. Abdomen light beige, without any pattern. Spinnerets uniformly beige. Measurements of legs: I: 12.10 (3.41, 2.20, 3.00, 2.90, 0.59), II: 10.90 (3.06, 1.93, 2.60, 2.65, 0.66), III: 7.87 (2.27, 1.25, 1.67, 2.05, 0.63), IV: 11.05 (3.24, 1.65, 2.43, 2.96, 0.77). Spination: I, II: no spines. III: Ti: 2pl, 2rl, 2v; Mt: 3pl, 2rl, 6v. IV: Fe: 3d; Ti: 2rl, 2v; Mt: 3pl, 3rl, 6v.

Endogyne as in Fig. 7; receptacle (*Re*) with posteriorly bent lateral edges (*Rl*), ca 8 times longer than wide and as wide as transverse bar (*Tb*); dorsal arch (*Da*) ca 2.5 times wider than long, with rounded anterior edges; transverse bar (*Tb*) long and thin, ca 15 times longer than wide; posteriorly with pair of diverticula (*Pd*).

##### Note.

The holotype of *D.nakhchivanica* has four teeth on the median process (*Mp*), while Armenian specimens have 5–7. We have tentatively considered this as an intraspecific variation. Certain variations in the male palp, some of which depend on the photography angle, are illustrated in Figs 4A and 6A–D.

##### Distribution.

Azerbaijan (Nakhchivan) and Armenia (new record) (Fig. 10).

### ﻿Subfamily Harpacteinae Cooke, 1965

**Genus *Harpactea* Bristowe, 1939**

#### ﻿ Harpactea armenica

Taxon classificationAnimaliaAraneaeDysderidae

Dunin, 1989

F1F60027-7445-56EF-968F-BDD4C67717A7

Figs 8A, B , 9A–D

Harpactea armenica Dunin, 1989: 142, figs 1–3 (♂♀).
Harpactea armenica : Dunin 1992: 68, fig. 13 (♂♀).

##### Materials examined.

Armenia: *Aragatsotn Province*: 24♂ 34♀ 29 juv. (ZMMU), foothills of Aragates Mt., 40°23'N, 44°13'E, 2200 m, 16.05.2021 (Y.M. Marusik); *Ararat Province*: 1♂ (ZMMU), env. of Urtsadzor Vill., 39°55'26"N, 44°48'53"E, 1040 m, 6.05.2021 (Y.M. Marusik); 1♂ 2♀ (ZMMU), env. of Urtsalanj Vill., 39°49'N, 44°59'E, 1800 m, 11.05.2021 (Y.M. Marusik); 5♂ 1♀ (ZMMU), Garni gorge, Azat river, 40°06'32"N, 44°43'57"E, 1240 m, 17.05.2021 (Y.M. Marusik); *Kotayk Province*: 3♂ 2♀ (ZMMU), env. of Geghadir, 40°09'N, 44°38'E, 15.05.2021 (Y.M. Marusik); 2♂ 4♀ (ZMMU), env. of Solak Vill., 40°28'24"N, 44°42'57"E, 14.05.2021 (Y.M. Marusik); 1♂ 1♀ (ZMMU), env. of Aghveran Vill., 40°29'54"N, 44°35'24"E, 7–8.05.2021 (Y.M. Marusik); 5♂ 7♀ 4 juv. (ZMMU), same locality, 7.05.2021 (Y.M. Marusik); *Vayots Dzor Province*: 21♂14♀ (ZMMU), Shatin, 39°50'N, 45°19'E, 9.05.2021 (Y.M. Marusik); 2♂ 3♀ (ZMMU), Shatin, 39°50'N, 45°19'E, 9.05.2021 (Y.M. Marusik); *Yerevan*: 7♂ 9♀ (ZMMU), Yerevan, botanical garden, 40°12'43"N, 44°33'21"E, 17.05.2021 (Y.M. Marusik). Iran: *East Azerbaijan Province*: 1♂ 3♀ (MHNG), NE of Sofian, 38°21'N, 45°50'E, 5.06.1975 (A. Senglet); *Gilan Province*: 1♂ 3♀ (MHNG), Asalem, 37°45'N, 48°57'E, 11.06.1975 (A. Senglet); 1♂ 3♀ (MHNG), Lahidjan, 37°11'N, 49°54'E, 5.07.1973 (A. Senglet); *Mazandaran Province*: 1♂ 3♀ (MHNG), road to Djavaherdeh, 36°55'N, 50°33'E, 1200 m, 7.08.1974 (A. Senglet); *West Azerbaijan Province*: 1♂ 3♀ (MHNG), Qara Kelisa, 39°04'N, 44°38'E, 31.05.1975 (A. Senglet).

##### Comments.

This species is most similar to *H.secunda* Dunin, 1989, a species restricted to northern Armenia, but can be differentiated from it by the base of embolus (*Eb*) (slightly and roundly bent, vs sharply bent to about 70°). It is possible that this small difference is only an intraspecific variation; in order to verify this, it is necessary to collect additional material from northern Armenia or examine the type specimens of *H.secunda*. Furthermore, it is likely that the female illustrated by Dunin (1989, 1992) belongs to another species. All females collected in Armenia and Iran examined here have a longer receptacle, longer than anterior diverticulum (*Ad*) *sensu*Dunin (1992), and lack bulbous thickening of the receptacle (*Re*, Fig. 9D).

**Figure 8.**
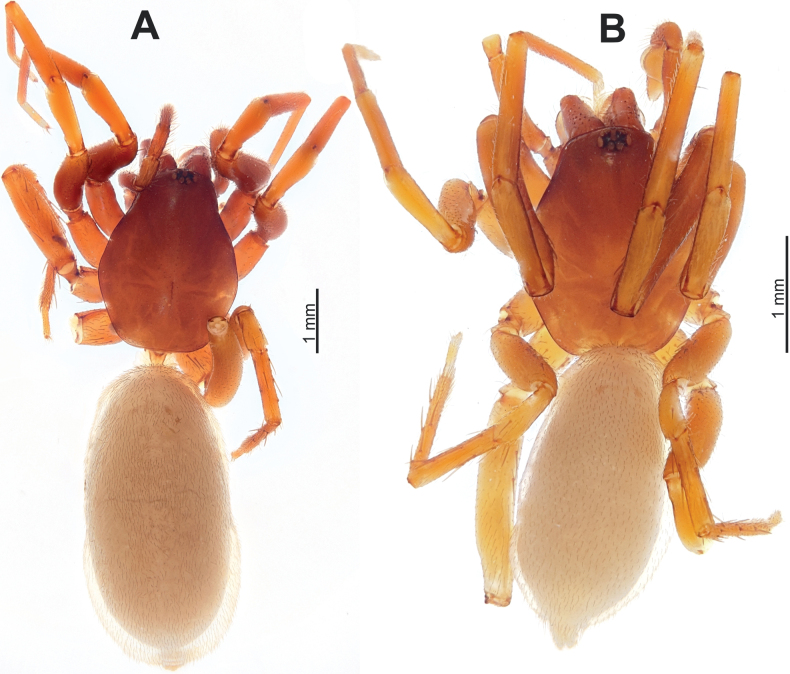
Habitus of *Harpacteaarmenica*, male (**A**) and female (**B**), dorsal.

**Figure 9.**
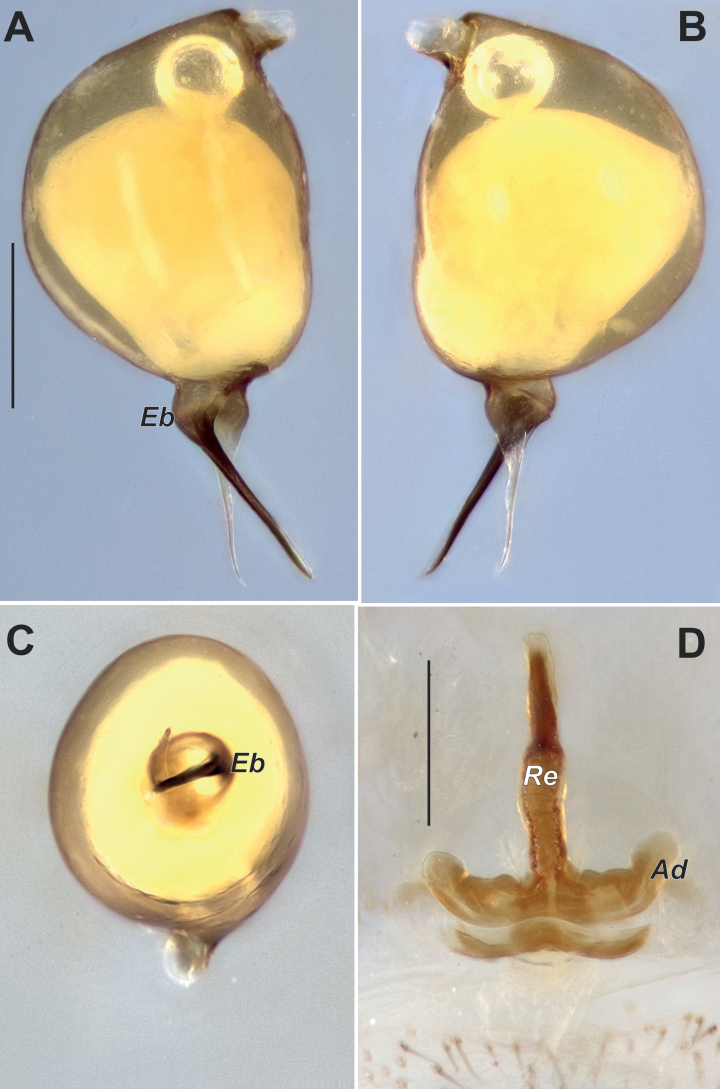
Copulatory organs of *Harpacteaarmenica***A–C** bulb, prolateral, retrolateral and ventral **D** endogyne, dorsal. Abbreviations: *Ad* – anterior diverticulum, *Eb* – base of embolus, *Re* – receptacle. Scale bars: 0.2 mm.

##### Distribution.

**Figure 10.**
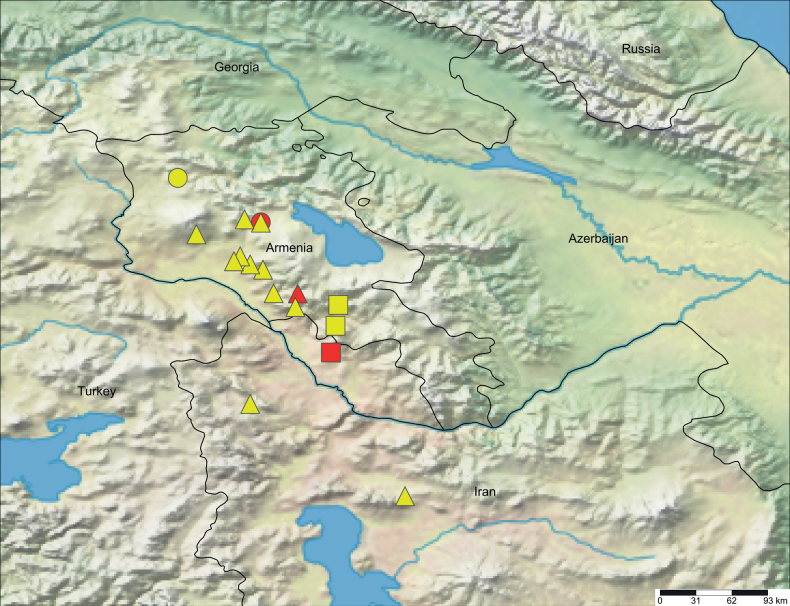
Distribution records of *Dysderahaykana* sp. nov. (circles), *D.nakhchivanica* (squares), and *Harpacteaarmenica* (triangles). Type localities are marked with red.

This species was previously known from only two localities in Armenia: Yerevan and the southernmost part of Kotayk Province. The new records slightly extend the known range of the species to the north, approximately 5° to the east, and around 4° to the south. Additionally, this species is newly recorded from Iran (Fig. 10).

## Supplementary Material

XML Treatment for Dysdera haykana

XML Treatment for Dysdera nakhchivanica

XML Treatment for Harpactea armenica

## ﻿Additional information

### Author ORCIDs

Armine Kosyan https://orcid.org/0000-0001-5692-5369

Alireza Zamani https://orcid.org/0000-0002-8084-9666

Yuri M. Marusik https://orcid.org/0000-0002-4499-5148

### Data availability

All of the data that support the findings of this study are available in the main text.
